# Estuarine crocodiles in a tropical coastal floodplain obtain nutrition from terrestrial prey

**DOI:** 10.1371/journal.pone.0197159

**Published:** 2018-06-06

**Authors:** Maria Fernanda Adame, Timothy D. Jardine, Brian Fry, Dominic Valdez, Garry Lindner, Jonathan Nadji, Stuart E. Bunn

**Affiliations:** 1 Australian Rivers Institute, Griffith University, Nathan, QLD, Australia; 2 School of Environment and Sustainability, University of Saskatchewan, Saskatoon, SK, Canada; 3 Kakadu National Park, Jabiru, NT, Australia; Charles Darwin University, AUSTRALIA

## Abstract

The estuarine crocodile (*Crocodylus porosus*) is one of the largest and most widespread crocodilians in the world. Although considered an apex species, the role of the estuarine crocodile in aquatic foodwebs is poorly understood; we know what crocodiles ingest, but not what nourishes them. In this study, we used a combination of stable isotope measurements (δ^13^C, δ^15^N, and δ^34^S) and direct feeding observations to identify the source of nutrition of estuarine crocodiles in Kakadu National Park, Northern Australia. Our results show that most crocodiles sampled (size 850 – 4200mm, with 76% of them being > 2.5 m) consume a large variety of prey, however a large proportion of their nutrition is derived from terrestrial prey. Introduced species such as water buffaloes (*Bubalus bubalis*) and pigs (*Sus scrofa*) could contribute between 53 and 84% to the nutrition of the sampled crocodiles. The isotopic composition of large crocodiles (total length > 3 m) suggested possible increase in marine prey consumption with size (*R*^2^ = 0.30; *p* = 0.005). Additionally, we found crocodiles sampled in the dry season had on average higher terrestrial contributions compared to crocodiles sampled during the wet season (84.1 ± 2.4% versus 55.4 ± 7.0%). Overall, we found that terrestrial prey are important source of nutrition for many crocodiles in this region where introduced herbivorous mammals are abundant.

## Introduction

The estuarine crocodile is an iconic and widespread predator in the tropics, occurring from southern India to Northern Australia [1]. The estuarine crocodile is one of the largest living crocodilians, reaching 7m in length. It has a reputation of being aggressive, feeding on a wide variety of prey that can include humans [1–3]. The diet of the estuarine crocodile is highly variable among populations and organisms, and commonly includes ontogenetic shifts [4–6]. In Australia, juveniles and adult crocodiles feed on low trophic levels, while medium-sized individuals feed on higher trophic levels [7]. Stomach contents of estuarine crocodiles in Australia and Malaysia show that juveniles feed on crabs, prawns, insects, and small fish, while sub adults (120–180 cm total length, TL) feed on a mixture of crustaceans, mammals, and birds [6,8–10]. The diet of adults (TL > 180 cm) has been mainly inferred from direct observations and includes a wide range of riverine, terrestrial, and marine organisms. The data available show that estuarine crocodiles ingest a large variety of foods, however, which prey are the most important resource for their nutrition is still unknown.

Contrary to direct observations of feeding activity or stomach contents, stable isotopes help discern assimilated prey that contributes to nutrition rather than prey that is simply ingested [11]. For example, because plant material has been observed in the stomachs of crocodiles, we could falsely infer that crocodiles feed on plants, an unlikely source of nutrition for a carnivorous predator [8]. Analyzing the gut contents of large, aggressive crocodiles is difficult because animals are hard to capture and subdue. Thus, many studies have concentrated on studying smaller individuals [6,8,9]. It is important to include adult individuals in studies of crocodile diets, because they comprise the majority of organisms in healthy populations [12]. The combination of direct dietary observations with ecological tracers can help identify the diet and nutrition sources of predators that are difficult to sample.

The diet of a top predator has implications at the ecosystem level. For example, adult *Alligator mississippiensis* inhabit freshwaters, but consume substantial amounts of marine prey; thus, they play an important role in the exchange of nutrients and carbon between freshwater and marine ecosystems [10,13]. Another species, the Australian freshwater crocodile, *C*. *johnstoni*, has a diet consisting mostly of aquatic prey, such as fish, and crustaceans [14,15]. Therefore, *C*. *johnstoni* could be important in regulating the local freshwater food web. The diet of predators has cascading effects onto ecosystem functions, such as in nutrient cycling and carbon sequestration [16–18]. Understanding the diet of crocodiles can provide information on their role in nutrient transport, top-down regulation of prey populations, and terrestrial-marine connectivity.

The role of crocodiles in aquatic and terrestrial ecosystems can be disrupted (e.g. [19]). For example, in northern Australia, introduced terrestrial species are abundant, with a population of water buffalo (*Bubalus bubalis*) currently numbering more than 150,000; and with feral pigs (*Sus scrofa*) reaching 10–20 individuals per square kilometer [20]. As a result, prey biomass of introduced mammals is large and vastly exceeds that of native mammals in the region [21]. Currently, the population of estuarine crocodiles in Northern Australia is growing [12]. After hunting was banned in the 1970s, crocodile numbers have recovered from an estimated 3,000 in 1984 to 70,000 in 1998 [12]. It is unknown whether these two factors, abundant terrestrial prey and a growing population, are somehow related.

**Fig 1:**
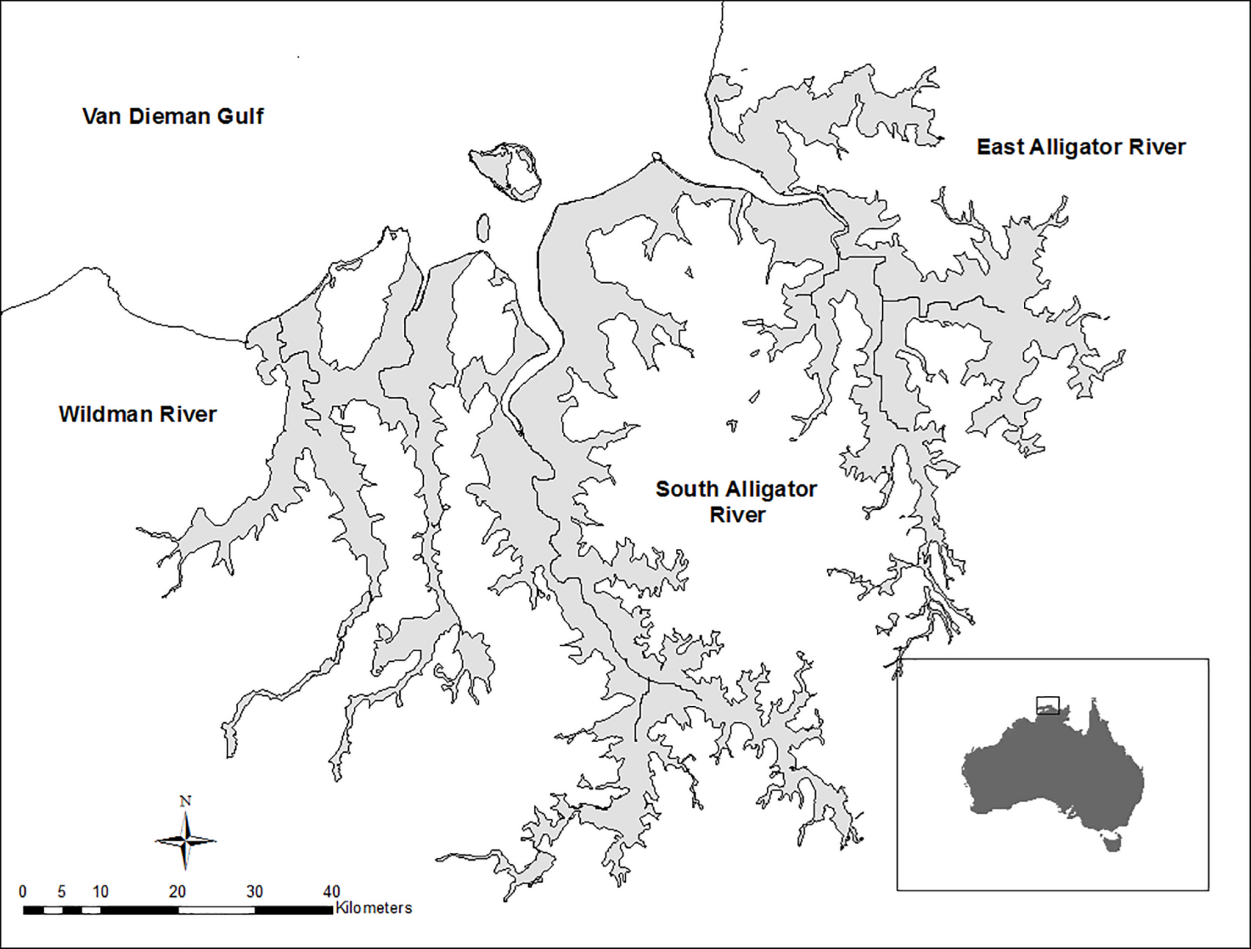
Sampling sites within rivers Kakadu National Park, Northern Territory, Australia. Crocodiles were sampled in three rivers and their respective floodplain areas (grey): Wildman, South and East Alligator Rivers.

In this study, we use a combination of stable isotope values (δ^13^C, δ^15^N, and δ^34^S) of crocodiles and their potential prey to identify the diet and source of nutrition of a large and growing population of estuarine crocodiles in Northern Australia. We included a large number of individual crocodiles (n = 45) ranging from juveniles of 85 cm TL to adults of 4.2 m TL. We sampled potential prey from the rivers (barramundi, *Lates calcarifer*, and mullet, *Liza ordensis*), land (wallaby, *Macropus agilis*; buffalo, *B*. *bubalis;* and feral pigs, *S*. *scrofa*), and ocean (giant sea catfish, *Netuma thalassina*). We also included direct field observations of feeding behavior and gut contents. Our study sites are within three rivers in Northern Australia (Fig 1) which maintain intact hydrology and have large and highly productive seasonal floodplains [22,23]. The main goals of the study were: first, to identify the sources of nutrition of the estuarine crocodile population in Kakadu National Park, Northern Australia, and second, to investigate how introduced and abundant terrestrial prey may have affected the ecological role of this iconic predator in the region.

## Materials and methods

### Study site

All handling of animals was conducted under Griffith University animal’s ethics protocol approved by the Animal Ethics Committee (ENV/08/11/AEC) and Kakadu National Park permit guidelines (RK 786). Sampling was conducted in Kakadu National Park in northern Australia. Samples were collected from the Wildman River (WR), South Alligator River (SAR) and East Alligator River (EAR) all of which have undisturbed flooding regimes (Fig 1). The rivers are bounded by the Van Diemen Gulf to the north and the Kakadu escarpment to the south. The region is a tropical savannah characterized by a monsoonal climate with two distinct seasons, wet and dry. The wet season usually starts between October and December, typically lasting three to four months. During this period, the region receives 1,300–1,500 mm of rain [24], which results in the inundation of the floodplains (Fig 1)

### Sample collection

Sampling was conducted from 2012 to 2014. Crocodiles were opportunistically sampled in the SAR, EAR, and the WR. Most samples were obtained from the crocodile management tagging program that surveys the crocodile population and distribution throughout Kakadu National Park. These crocodiles were trapped, measured and released for monitoring purposes. Most of the individuals sampled through this program (44%) are around 2.5 m, which is the most common crocodile size in the park (44%, G. Lindner pers. comm). Other samples were obtained from crocodiles that were relocated due to their overt interest towards people or boats, or were identified as nuisance animals that needed to be removed from the local community or tourist locations. A few samples were obtained from traps in the upper reaches of rivers, from dead crocodiles that were killed by other aggressive crocodiles, or by vehicles on the road.

Each sample consisted of a piece of scute taken from the mid-section of the tail (S1 Fig). Scutes are made of a combination of keratin and collagen [5]. Our samples consisted predominantly of keratin. Similar to other studies [25], we found no significant difference between the isotope value of keratin and collagen (D. Valdez, unpublished data). When possible, the total length (TL) of the crocodile was measured (n = 25) and sex was determined by probing the cloaca with a finger. We sampled 45 crocodiles that ranged from 85 cm to 4.2 m TL, from which 14 male and 5 female individuals were identified (S1 Table). Crocodiles were sampled during the dry (n = 20) and the wet season (n = 25). We sampled 31 crocodiles from the EA, 9 from the SA, and 2 from the WR. We also analyzed samples of 3 individuals from unknown locations within the National Park (S1 Table).

We classified potential prey as terrestrial, riverine, riverine-marine, or marine. Terrestrial prey included feral pigs (*S*. *scrofa*, *n* = 58), wallaby (*Macropus agilis*, n = 35), and buffalo (*B*. *bubalis*, *n* = 8); riverine prey were represented by mullet (*L*. *ordensis*, *n* = 258); riverine-marine by barramundi (*Lates calcarifer*, *n* = 385); and marine prey by the giant catfish (*Netuma thalassina*, *n* = 5). Muscle tissue from terrestrial animals was opportunistically collected from road kill and from individuals killed as part of the management program of feral species of Kakadu National Park. Buffalo tissue samples were also collected from a nearby farm. Riverine and marine fish were collected using multiple methods including hand line, rod and reel, cast net, 10 and 16-cm gill nets that were 20m in length, and backpack and boat electro-fishers (Smith-Root, Inc. Vancouver, WA, and U.S.A.). For large fish, caudal fin tissue was sampled non-lethally for stable isotope analyses because fin tissue is a reliable surrogate for muscle tissue [26]. Other potential prey such as birds, reptiles, and sharks were opportunistically collected (S2 Table).

Tissue samples were rinsed and transported frozen. Samples were oven-dried at 60°C for 24 h before being ground to a fine powder and homogenised with a ball and mill grinder. Analyses for δ^13^C, δ^15^N, and δ^34^S were conducted with an elemental-analyser isotope ratio mass spectrometer system (EA-IRMS, Sercon System, Griffith University). Analytical errors, based on standard deviations of in-house standards, were < 0.1‰ for δ^13^C, < 0.2 ‰ for δ^15^N, and < 0.5‰ for δ^34^S.

To estimate the contribution of prey to the crocodile’s diet, we corrected the isotope values of the crocodile for isotopic discrimination that occurs during prey consumption. We subtracted 1.4 ‰ from δ^13^C and 3.0 ‰ from δ^15^N for each individual based on isotopic discrimination (Δ) of keratin in captive estuarine crocodiles in Northern Australia [7]. Because the trophic correction for wild crocodiles is uncertain, we assessed the effect of using a range of isotopic discrimination factors, including those estimated for other species of crocodilians (American alligator, *Alligator mississippiensis* and broad-snouted caiman, *Caiman latirostris*), which have been reported to be lower (Δ = 0.6 and 0.9‰ for δ^13^C, and 1.2 and 0.8‰ for δ^15^N, respectively [25,27]). We did not apply a correction to δ^34^S data because trophic isotopic discrimination of δ^34^S is minimal (Δ = 0.5‰, [28]).

Isotope turnover in crocodilians is likely to be slow, for example, in captive American alligators (*Alligator mississippiensis*), δ^13^C has a half-live of 142 days and δ^15^N of 277 days [25]. Thus, we expected difficulties in assessing changes in diet at seasonal time scales. However, wild estuarine crocodiles have food conversion rates twice as high as captive ones [6,29], which could translate into faster isotopic turnover rates. To test whether we could identify seasonal differences in the diet, we compared isotope values of crocodiles sampled during the wet versus crocodiles sampled during the dry season.

### Direct observations

We compiled observations of feeding activities from records held by National Park rangers of Kakadu National Park. Direct feeding of crocodiles is likely to be biased towards crocodiles hunting terrestrial prey in the water-edge. Gut contents were also recorded from individual crocodiles that were killed for management purposes.

### Data analyses

We tested for differences among isotope values with Analyses of Variance (ANOVA), where isotope value (δ^13^C, δ^15^N, and δ^34^S) was the dependent variable, and sex, catchment, and season were the independent and/or random factors of the model. We also tested for difference among isotopic values of prey (terrestrial, riverine, riverine-marine, and marine); when differences were significant, Bonferroni post-hoc tests were conducted. Linear regressions were conducted to assess the relation between isotope value and crocodile size, and between food source contributions and crocodile size. Normality was assessed with probability plots and Shapiro-Wilk tests. When the variable was not normally distributed (e.g. % food contribution), it was transformed (log_10_). The statistical tests were performed with SPSS Statistics (v21, IBM, New York, USA). Values reported are means and standard errors, unless specified.

The relative contribution of each prey group to the nutrition of the crocodiles was first assessed by plotting the mean values of crocodiles versus possible food sources. Isotope mixing models for interpreting feeding relations in animals are generally based on the assumption that important food sources are included and well-characterized, and that animals have isotope values that are intermediate between source values of included prey [30]. However, some individual crocodiles were not clearly intermediate between potential prey sources, so that some sources may have been missed in spite of multi-year sampling. Also source mixing implies that animals have some access to all sources, but for crocodiles that can vary widely in their residency and mobility patterns, access to all sources did not seem assured [31]. The possibility of missing sources and lack of access to other sources led us to use two different but related isotope modelling approaches for interpreting crocodile diets.

The first approach used mean isotope values and proximity of crocodile and source values to assess similarity or affinity. This “proximity” technique was developed in the 1990s (Reviewed in [32]) and gives results generally similar to mixing models. The technique differs from mixing models in that it does not require that animals are intermediate between source values, and gives a somewhat more qualitative estimate of similarity or affinity between samples and potential sources, somewhat like cluster analysis results. This looser similarity assessment is more consistent with our sampling strategy and limited knowledge of crocodile behaviour. This approach can give biased results if a nearby source is split into several similar groups, with a result that the new cluster of sources acquires an inflated importance. Recognizing this problem, we were careful to not split sources into too many categories in our proximity analyses. In this proximity approach, distance from the crocodile mean to each source was calculated in 3D isotope space using Pythagorean distances:

$$d_{i}=\sqrt{\left[(\delta^{13}C_{croc}-\delta^{13}C_{source_{i}})+(\delta^{15}N_{croc}-\delta^{15}N_{source_{i}})+(\delta^{34}S_{croc}-\delta^{34}S_{source_{i}}\right)}]$$ Eq 1

where *d*_*i*_ is the Euclidean distance from the crocodile value to the value of the *i*^th^ source. The distances to all sources were inverted and summed, then the inverted distances divided by this sum and multiplied by 100 to give percent estimates of each source value. Results from this inverse distance or proximity approach were used to understand population-level mean trends in crocodile feeding. We performed two separate analyses, one for the EAR and one for the SAR. Due to low number of samples for the WR (n = 2), the crocodiles from this river were not included in this analysis.

The second approach was to focus on individuals where sources were aggregated into four categories, with a standardized Bayesian mixing model (SIAR Solo) applied to each animal [33]. We did this because crocodiles have a wide variety of diets and tend to be individualistic in their diet choices [13]. Unknown and missed sources are inherently aggregated into four groups: terrestrial, riverine, riverine-marine, and marine. The application of SIAR Solo gave standardized profiles of resource use that often differed among individual crocodiles. SIAR and other Bayesian mixing models commonly have a bias towards “all sources are equal” or generalist solutions [34–36], so we used the SIAR Solo approach as a conservative way to find instances where individual differences were still apparent for crocodiles. To assess the effect of seasonality, we separated crocodiles sampled during the dry season from those sampled during wet season.

Overall, the first affinity approach was applied to means for a generalized overview of average crocodile feeding at the whole landscape level. The SIAR Solo mixing model approach probed for variations in resource use by individuals, with this Bayesian approach expected to identify the more robust feeding differences among individuals. We also applied the first proximity approach to individuals, finding that overall it gave similar indications of differences among individuals evident in the SIAR Solo approach. This congruence of results from the proximity and Bayesian approaches has also been previously observed [37].

## Results

Direct observations of crocodile feeding activities confirmed that crocodiles consumed a wide variety of organisms (Table 1). Crocodiles in the study area were observed to feed on cattle, pigs, water buffalo, as well as flying fox (*Pteropus* sp) and snakes (brown snake, *Pseudonaja textilis*, and pythons). They are also known to feed on dogs and humans, with four fatal attacks on humans in the region since 1978. Crocodiles fed on birds including magpie goose (*Anseranas semipalmata*), heron (*Ardea sp*), cormorants (*Phalacrocorax* sp), egrets (*Egretta sp*), ibis (*Threskiornis moluccus*), and spoonbills (*Platalea sp*). Riverine prey included mullet and barramundi. Finally, crocodiles were observed to consume marine prey, including prawns, octopus, flatback turtle (*Natator depressus*) and turtle hatchlings. Stomach contents of crocodiles and specific sightings from this study are shown in Table 1.

**Table 1:** Observations of feeding habits and stomach content of crocodiles in Kakadu National Park.

Crocodile size (m)	Prey	
Stomach content		
5.0	R	catfish
4.8	T	human remains
4.6	T / R	human, fish bones
4.6	T	dog
4.4	T / M	grasshopper, fish bones, mangrove leaves, grass, rocks, bullet remains
4.4	T	echidna
4.1	T	human remains, pig meat, plastic
4	T	wallaby, pig
4	T	freshwater turtle
4	T / M	mud crabs, pig hair
3.6	T	Human and crocodile remains, pig leg, bird nails, grass
3.5	T	flying fox
3.0	T / R	bream, barramundi, fish bones, feathers, plants, rocks
2.8	M	hawksbill turtle
2.5	T	goose feathers, dog
2.4	T	human remains, pig meat
2.2	T	pig meat
2.2	T	cane toad (*C*. *porosus*)
2.2	T / M	grasshopper, prawn
Direct feeding observations		
5	T	bull
4–5	T	cattle
4.5	T	horse
4.5	T	water buffalo
4.4		crocodile
4.2		crocodile remains
4.2		crocodile remains
>4	T	pigs
4	T	horse
4	R	water python
3.8	M	dolphin
3.5		crocodile
3.5	M	bull shark
3.0	T	pig

The origin of the prey is denoted by R = riverine; T = terrestrial; M = marine.

**Fig 2:**
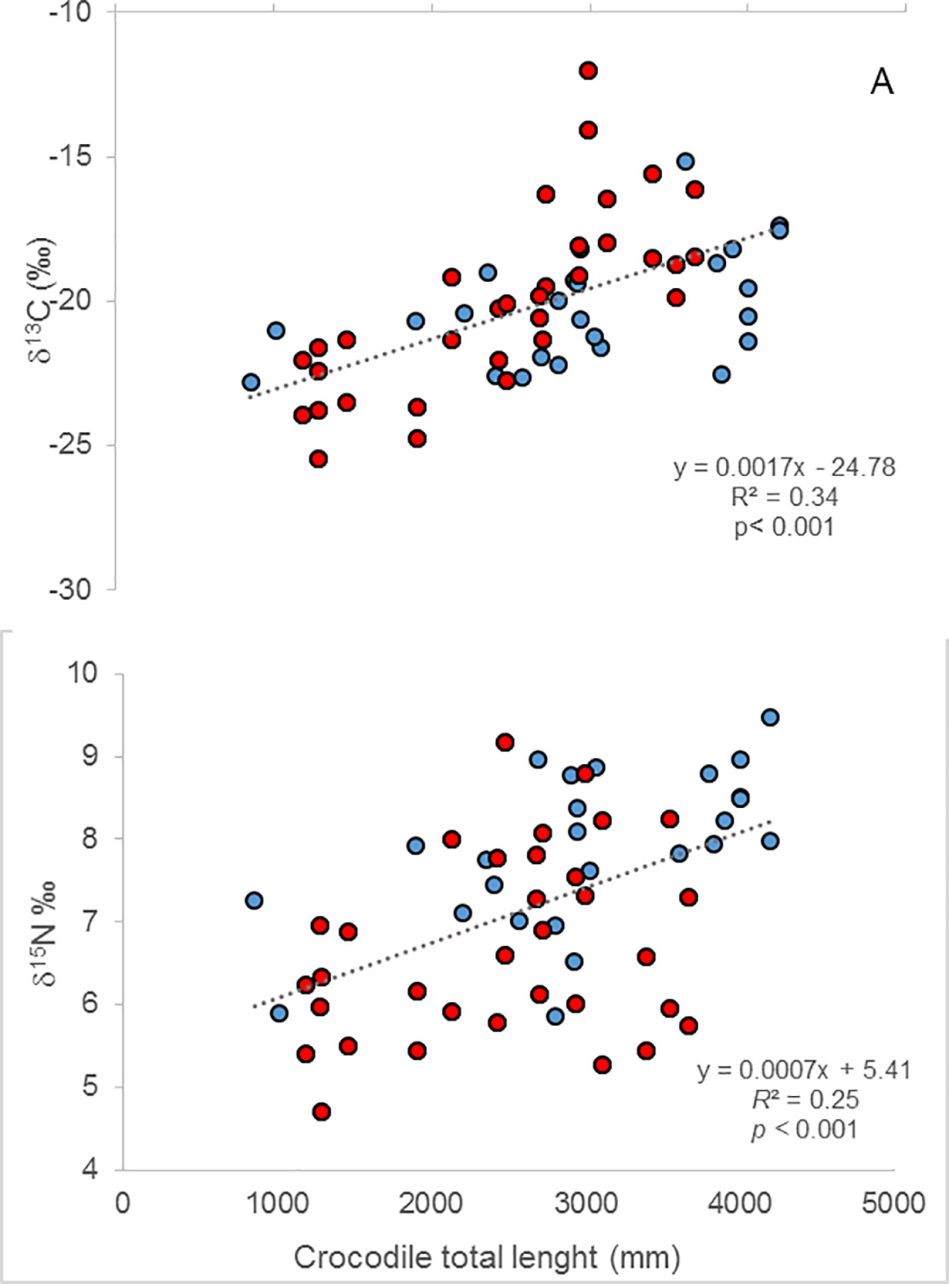
Correlation between isotopic value of crocodile scute (δ^13^C and δ^15^N; ‰) and body size (mm). Red circles are values from this study, blue circles are values from *C*. *porosus* in Cape York, Australia, from Hanson et al. 2015 [7]. Both datasets follow a linear trend of higher isotope values with increasing total body length (mm).

Crocodiles had a wide range of isotopic values (Table 2). The δ^13^C and δ^15^N values of crocodile were similar between catchments, season and sex of the crocodiles (catchment*season*sex *F*_1, 31_ = 0.93, *p* = 0.34; *F*_1, 31_ = 1.50, *p* = 0.23; and *F*_1, 31_ = 0.74, *p* = 0.79, respectively). However, there were significant differences in δ^34^S values between sexes in different seasons (sex*season *F*_2, 2_ = 69.2, *p* = 0.028). Finally, δ^13^C and δ^15^N, but not δ^34^S, significantly increased with body size (*R*^*2*^ = 0.22, *p* = 0.01; *R*^*2*^ = 0.35, *p* < 0.01; *R*^*2*^ = 0.05 *p* = 0.08, respectively; Fig 2), similar to data of *C*. *porosus* in other regions of Australia [7] (Fig 2)

**Table 2:** Mean ± se (min-max) of δ^13^C, δ^15^N and δ^34^S values of crocodiles and potential prey from Kakadu National Park, Australia.

	δ^13^C (‰)	δ^15^N (‰)	δ^34^S (‰)
Estuarine crocodile (*C*. *porosus*)	-20.9 ± 0.4 (-28.2 to -15.2) *n* = 45	7.7 ± 0.1 (5.4–9.6) *n* = 45	6.6 ± 0.7 (-4.1 to 13.2) *n* = 45
Mullet (*L*. *ordensis*)	-27.9 ± 0.4 (-36.1 to -17.1) *n* = 82	6.8 ± 0.1 (3.9 to 8.7) *n* = 82	6.5 ± 0.7 (-5.6 to 18.6) *n* = 69
Barramundi (*L*. *calcarifer*)	-24.2 ± 0.2 (-30.8 to -17.2) *n* = 170	8.9 ± 0.1 (4.9 to 12.2) *n* = 170	6.1 ± 0.5 (-5.8 to 15.3) *n* = 99
Wallaby *(M*. *agilis)*	-16.7 ± 0.3 (-19.7 to -14.4) *n* = 19	2.8 ± 0.2 (0.6 to 5.2) *n* = 19	10.6 ± 0.7 (6.1 to 12.2) *n* = 6
Water buffalo *(B*. *bubalis)*	-20.5 ± 0.6 (-23.6 to -16.2) *n* = 8	5.7 ± 0.1 (5.1 to 6.8) *n* = 8	2.8 ± 2.8 (-7.5 to 9.5) *n* = 4
Pig (*S*. *scrofa)*	-24.2 ± 0.2 (-27.7 to -21.1) *n* = 57	5.4 ± 0.1 (2.8 to 6.9) *n* = 57	4.8 ± 2.6 (-12.1 to 12.3) *n* = 10
Giant sea catfish *(N*. *thalassina)*	-16.5 ± 0.6 (-18.9 to -15.2) *n* = 5	11.2 ± 0.4 (9.6 to 12.2) *n* = 5	15.4 ± 0.6 (13.7 to 16.8) *n* = 5

Values are uncorrected for trophic fractionation

The isotopic composition of the prey was significantly different for terrestrial, riverine, riverine-marine and marine animals for δ^13^C (*F*_198, 3_ = 55.77, *p* <0.001, riverine and river-marine different from marine and terrestrial), δ^15^N (*F*_198, 3_ = 70.85, *p* <0.001, all different), and δ^34^S (marine different from the rest, *F*_198, 3_ = 4.59, *p* = 0.004; S3 Table.

**Fig 3:**
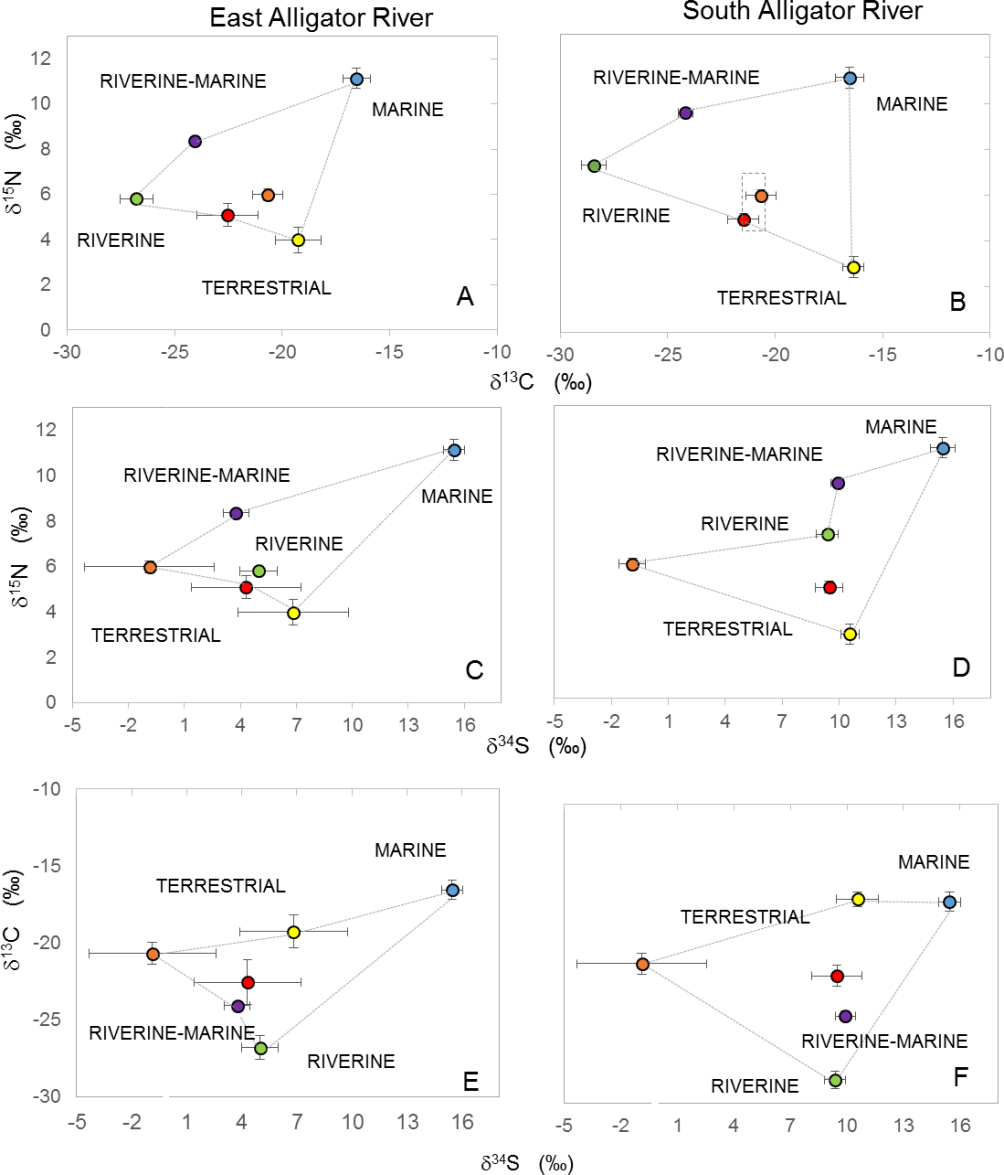
Isotopic composition (δ^34^S, δ^13^C and δ^15^N) of crocodiles (black diamonds) and potential prey. Samples were obtained fromthe East Alligator River (A,C, E) and South Alligator River (B,D,F), terrestrial prey includes water buffalo (orange circle), pigs (red circle) and wallabies (yellow circle); riverine is represented by mullet (green circle); riverine-marine prey is represented by barramundi (purple circle) and marine prey is represented by giant sea catfish (blue circle). Crocodile data were corrected to the level of prey by subtracting 1.4 ‰ from δ^13^C values [7]. The effect on the crocodile value of different fractionation factors is shown as a box of possible values around the crocodile mean in panel B.

**Fig 4:**
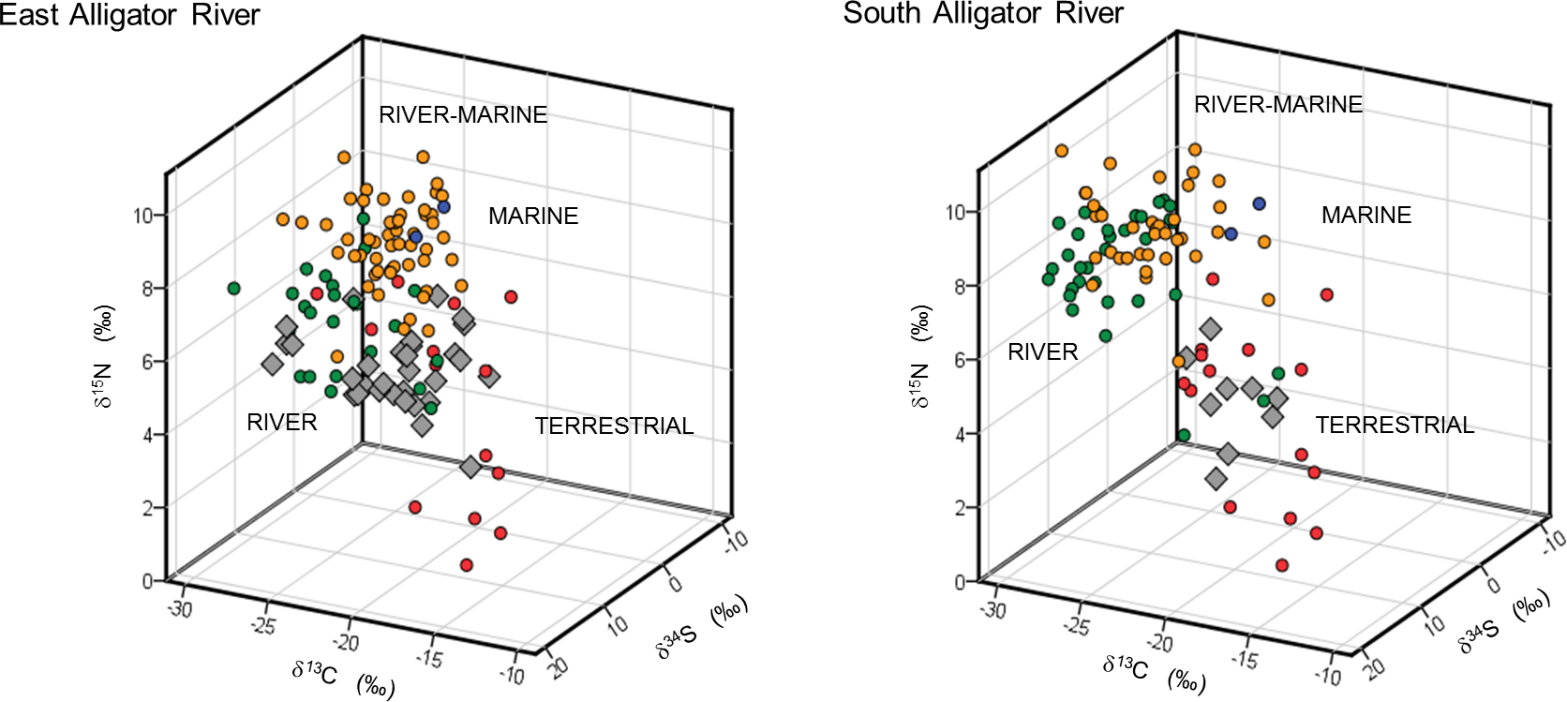
3D plot of the isotopic composition (δ^13^C, δ^15^N and δ^34^S) of crocodiles (diamonds) and potential prey. Samples are from the East and South Alligator Rivers; terrestrial prey includes water buffalo, pigs and wallabies; riverine prey is represented by mullet; riverine-marine prey is represented by barramundi and marine prey is represented by giant sea catfish.

Crocodile isotopic composition was closest to that of terrestrial animals in dual isotope space (δ^13^C vs δ^15^N, Fig 3A and 3B), especially pigs and buffaloes. When plotting δ^15^N vs δ^34^S, crocodiles in both EAR and SAR were closest to pigs and mullet (Fig 3C and 3D). In 3D isotope plots (Fig 4), crocodiles most closely plot around terrestrial and riverine sources. Using the proximity-based method for evaluating diet relationships in 3D isotope space (δ^13^C, δ^15^N, and δ^34^S), terrestrial animals had the highest contribution to the diet of crocodiles in both EAR and SAR with contributions of 58.9 ± 2.0 and 69.0 ± 0.6%, respectively (Fig 4). Contributions for pigs and buffalo were particularly high with 23.0 ± 1.2 and 22.2 ± 1.7% for the EAR, and 27.3 ± 2.9 and 24.0 ± 2.2% for the SAR. Contributions of other prey were similar between the EAR and SAR with 18.0 ± 1.8 and 9.2 ± 0.3% for riverine, 15.1 ± 0.5 and 12.0 ± 0.5% for riverine-marine, and 7.9 ± 0.3 and 9.7 ± 0.7% for marine prey.

When assessing the difference between different isotopic discrimination factors for δ^15^N, we found that if we used the minimum discrimination factor (Δ = 0.8 ‰) the contribution of river-marine prey increased by 28% (20.6 ± 0.9 and 16.9 ± 1.5% for EAR and SAR, respectively; Fig 3B). However, with either discrimination factor (minimum of 0.8 and maximum of 3.0‰), terrestrial prey was the dominant contributor to the crocodile’s diet (53.0 ± 1.7, and 58.6 ± 2.6% for the EAR and SAR, respectively), even when considering other possible prey such as birds, reptiles, crabs, shrimp, snails and sharks (S1 Table).

**Fig 5:**
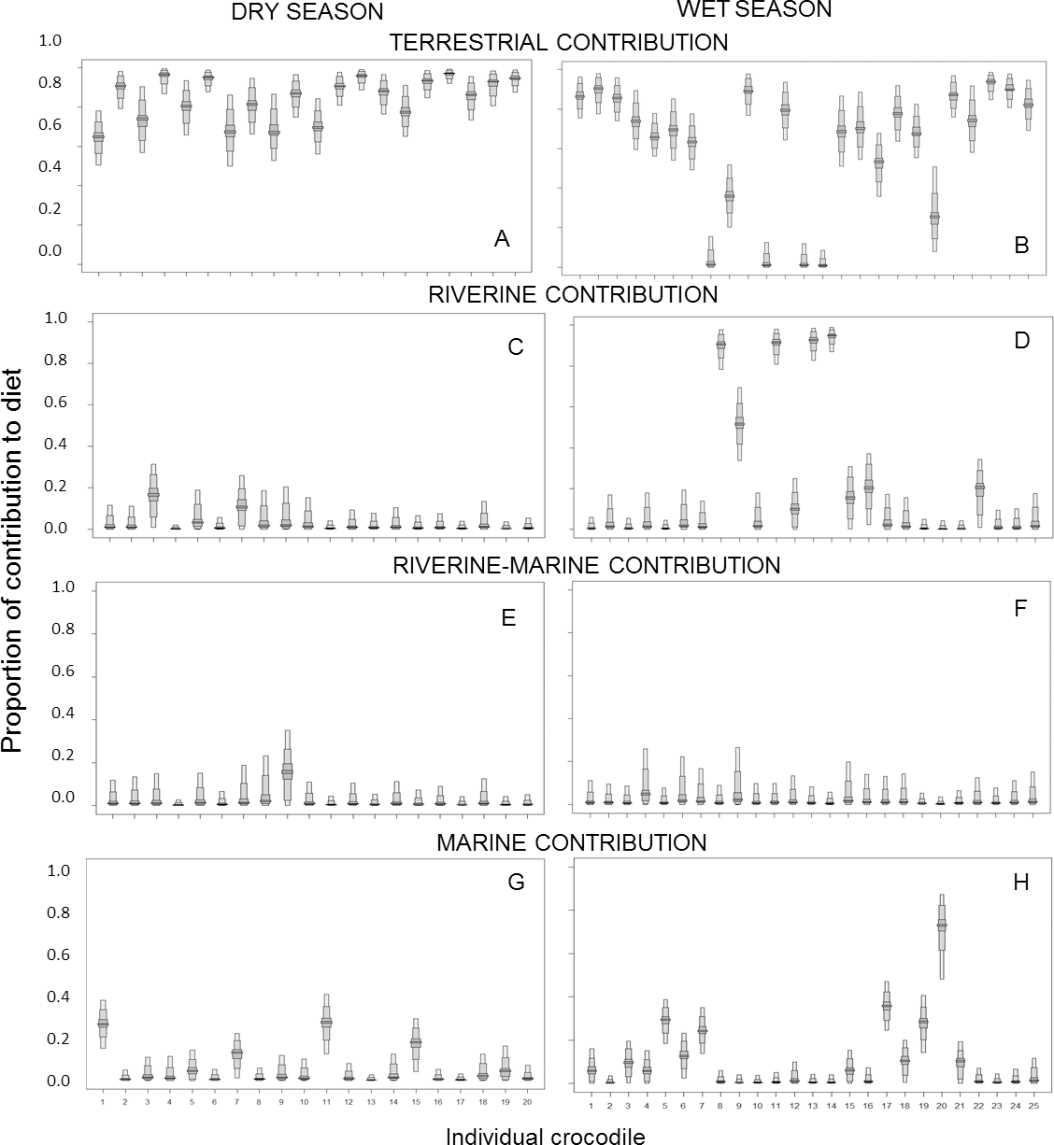
Proportion of contribution of prey to the nutrition ofestuarine crocodiles in Kakadu National Park. Crocodiles were caught during the dry and wet season and isotope values were analysed with SIAR solo. Terrestrial prey includes water buffalo, pigs and wallabies; riverine prey is represented by mullet; riverine-marine prey is represented by barramundi and marine prey is represented by giant sea catfish. Box plots represent the 5, 25, 75 and 95% credibility intervals.

The SIAR solo Bayesian model applied to individuals showed similar results, with terrestrial prey having the highest contribution, although values were higher with a mean of 72.9 ± 4.2%. Riverine prey had the second largest contribution with 14.4 ± 4.1%, followed by marine with 9.4 ± 4.3% and river-marine with 3.4 ± 0.7%. The stomach contents of one crocodile (number 23 in the wet season in Fig 5) were matched to its diet. This crocodile had wallaby, turtle and vegetation material in its stomach and isotope values that suggested a predominately terrestrial diet.

**Fig 6:**
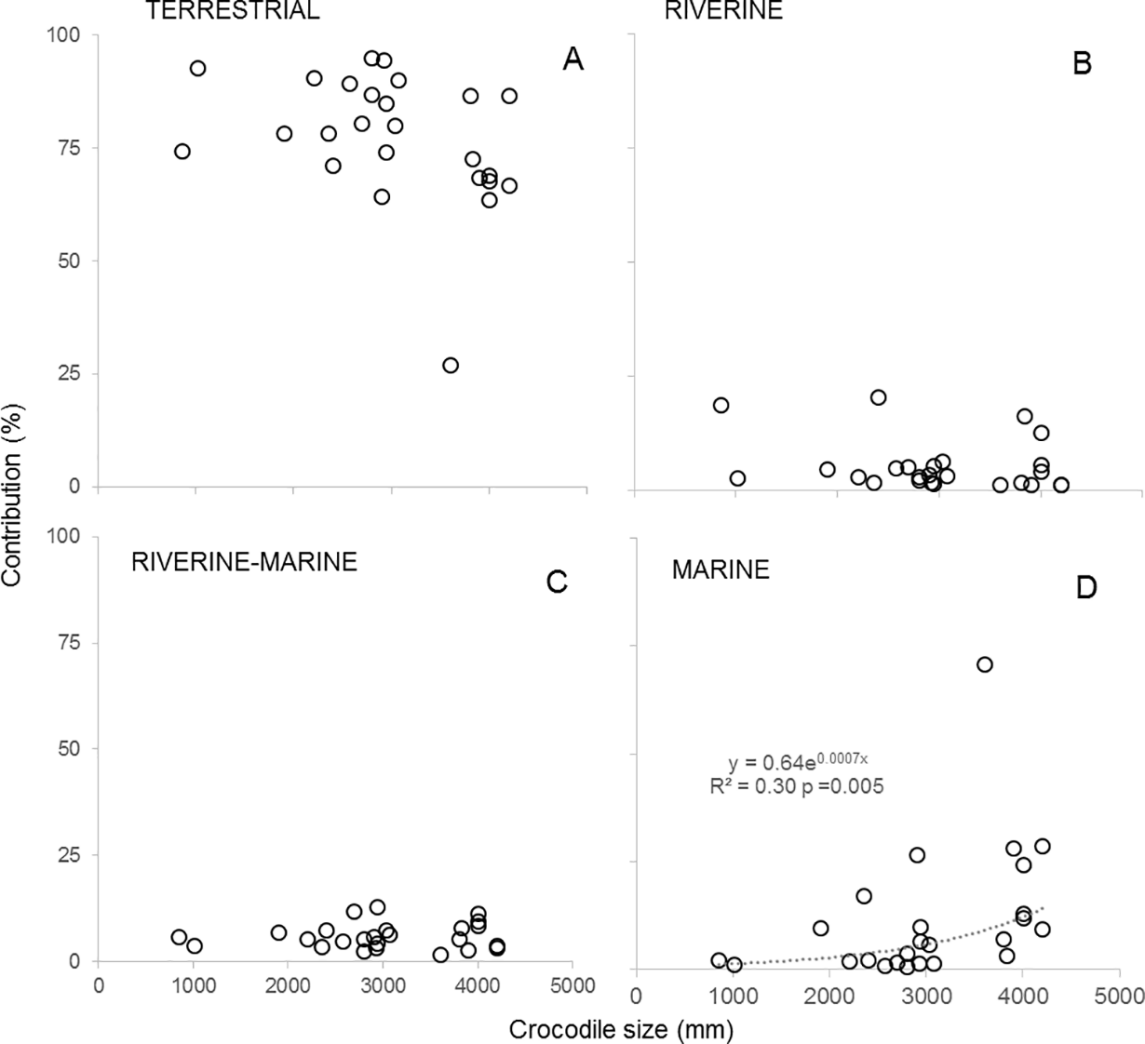
**Contribution of (A) terrestrial, (B) riverine, (C) riverine-marine and (D) marine prey to the diet of estuarine crocodiles.** The contribution was assessed with SIAR solo using data from crocodiles ranging from 0.85 to 4.2 m long (TL).

We found a difference in diet with crocodile size, with larger crocodiles having a higher contribution of marine prey to their diet compared to smaller ones (*R*^2 =^ 0.30; *p* = 0.005; Fig 6D). Additionally, the source of nutrition of crocodiles was variable between crocodiles sampled in different seasons (Fig 5). The crocodiles caught during the dry season had isotopic values that suggested a heavy reliance on terrestrial prey with a mean contribution of 84.1 ± 2.4%. The crocodiles caught during the wet season had a mean contribution of 55.4 ± 7.0% of terrestrial prey, 25.1 ± 8.1% of riverine prey, and 12.6 ± 3.9% of marine prey. However, this difference in wet versus dry season was mostly driven by six crocodiles, five individuals caught in the Magela floodplain in the EAR with predominately a riverine diet and one individual caught at Cahill’s crossing (EAR) which had a predominately a marine diet (Fig 5).

The proximity analyses for seasonal comparisons showed similar results than the SIAR Solo model, but with slightly different contributions. Terrestrial prey contributed 52% in the dry season versus 36% in the wet season; river prey contributed 19% in the dry season and 28% in the wet season; river-marine prey contributed 23% in the dry and 30% in the wet season; and finally, marine prey contributed 7% in the dry season and 6% in the wet season.

The difference in diet between the dry and wet season was also noted in the δ^15^N values, which were on average 0.6‰ higher in the wet compared to the dry season, a difference that was slight, but significant (*F*_1, 43_ = 4.28, *p* = 0.04). When considering sex and catchment in the model, the difference between seasons was not significant (season*sex*catchment *F*_1, 31_ = 1.50, *p* = 0.23). Nevertheless, low δ^15^N values seemed the best overall indication of dietary dependence on terrestrial prey in this study. Low δ^15^N values were found only in terrestrial herbivorous animals (pigs, buffalo and wallabies), but not in other vertebrates and invertebrates collected (birds, reptiles, shrimp, crabs, snails, shark, S1 Table).

## Discussion

Most estuarine crocodiles sampled in this study derived a large proportion of their nutrition from terrestrial prey. This result is surprising, because in general, crocodilians are considered apex aquatic predators (e.g. [1]). However, our results suggest that this population is having less influence in the aquatic food web than previously thought. Although estuarine crocodiles in this region consume a wide range of prey, it appears that for a large number of individuals, terrestrial animals are an important source of nutrition.

Previous studies have shown that terrestrial animals could provide an important source of nutrition for the estuarine crocodile in the region [6,38]. For example, native rats (*Rattus colletti* and *Xeromys myoides*) have been found in the stomachs of juvenile crocodiles (0.9–1.3 m TL [6,38]). Larger crocodiles (> 3m), are also known to hunt and kill large pigs and cattle at the water edge (G. Lindner pers. comm), and smaller individuals have been observed feeding on pig and cattle carrion left by larger dominant males [39] (G. Lindner, pers comm.). An estuarine crocodile requires approximately 4% of their body weight every week to maintain its body mass [6]. As such, a large crocodile of 1,000 kg would need 40 kg of food per week. Feral pigs weigh between 50–100 kg; thus, many crocodiles could take advantage of this prey that is large, abundant, and relatively easy to catch to satisfy their dietary requirements.

Despite the reliance on terrestrial prey for most of the sampled crocodiles, we found a difference in diet associated with body size. Previous results have suggested ontogenetic changes in the diet of estuarine crocodiles in Australia [7]. Our results support this finding; we found a significant increase in δ^13^C and δ^15^N values with body size. The difference in isotopic composition appears to be associated with higher marine contribution in the large crocodiles (3 to 4.5m TL). Similar ontogenetic shifts in diets have been found in other crocodilians such as *Crocodylus niloticus* in Botswana [5], *Alligator mississippiensis* in the US, and *Crocodylus acutus* in Belize; similar to our results, adults of the latter species consume more marine prey than juveniles [13,40]. However, the difference in δ^13^C and δ^15^N values could also mean different metabolic processes associated with body size [41].

Crocodiles are highly individualistic in their diets [13,42], which is reflected in the wide range of isotopic values within their tissues. Large variability in isotope values from crocodiles also suggests a wide diversity of habitats, diversity of prey available, and different home range strategies [31,43]. For instance, some crocodiles are highly mobile (e.g. travelling 1000 km in 6 months), while others are fairly resident within their home ranges [15]. It has been hypothesized that differences in homing strategies result in differences in diet [31]. In our dataset, five crocodiles were caught in the inundated floodplains during the wet season [44]. These crocodiles could be nomadic individuals traveling onto the floodplain and taking advantage of its high productivity [45]. These individuals had primarily a riverine diet. Contrary, all the individuals sampled in the dry season had primarily a terrestrial diet. During the dry season, crocodiles are concentrated in waterholes along with terrestrial animals, including introduced pigs and buffaloes, which depend on these waterholes for drinking water [20]. During this period, terrestrial animals seem to provide abundant and relatively easy prey for these crocodiles that are hunting at the land-water interface [46].

Crocodile populations have declined in many areas of the world. In South Africa, a decline in population numbers of *C*. *niloticus* has been associated with water pollution [47]; in northern Australia, the decline of the freshwater crocodile, *C*. *johnstoni*, was related to the introduction of the poisonous cane toad (*Bufo marinus*. [19]).The estuarine crocodile population in Kakadu National Park appears to be stable [12,48], with individuals having a wide trophic niche and large geographical distribution. It seems plausible that the capacity of the crocodiles to feed on introduced terrestrial prey has played a role in the recovery of their population [49,50], probably because the carrying capacity of their habitat has increased due to the increase in prey, particularly pigs. Crocodiles appear to be exerting top-down pressure on pigs and buffaloes, which are considered serious pests in the area. Our results provide an empirical example of a native predator adapting to an invasive prey and capitalizing on it [51]. A similar phenomenon might be occurring in other areas where introduced terrestrial prey has become abundant. For example, estuarine crocodiles in the Gulf of Carpentaria (northeast Australia) also have relatively low δ^15^N values (2 to 5‰) and high δ^13^C values (-26 to -18‰, Fig 2 [7]). These values are similar to those in the current study, and are consistent with the isotopic composition of terrestrial herbivores.

## Conclusion

Based on our isotopic analyses and direct observations, we found that estuarine crocodiles in Kakadu National Park, Northern Australia consume a large variety of prey. However, most of the crocodiles sampled in this study derive a large proportion of their nutrition from terrestrial prey.

## Supporting information

S1 Fig Crocodile scute sampled for isotope analyses of a *Crocodylus porosus* individual. (TIFF)

S1 Table Characteristics of *Crocodylus porosus* individuals from in Kakadu National Park, Australia. EAR = East Alligator River; SAR = South Alligator River, WR = Wildman River; M = Male; F = Female.
(DOCX)

S2 Table Isotope values of potential prey for estuarine crocodiles (*Crocodylus porosus*) in Kakadu National Park, Australia. Values are mean ± standard error.
(DOCX)

S3 Table Isotope values of potential prey for estuarine crocodiles (*Crocodylus porosus*) in Kakadu National Park, Australia: Marine, marine-riverine, riverine and terrestrial. (XLSX)
