# Correction: Estuarine crocodiles in a tropical coastal floodplain obtain nutrition from terrestrial prey

**DOI:** 10.1371/journal.pone.0200983

**Published:** 2018-07-16

**Authors:** Maria Fernanda Adame, Timothy D. Jardine, Brian Fry, Dominic Valdez, Garry Lindner, Jonathan Nadji, Stuart E. Bunn

Fig 3 is incomplete. The authors have provided a corrected version here.

**Fig 3:**
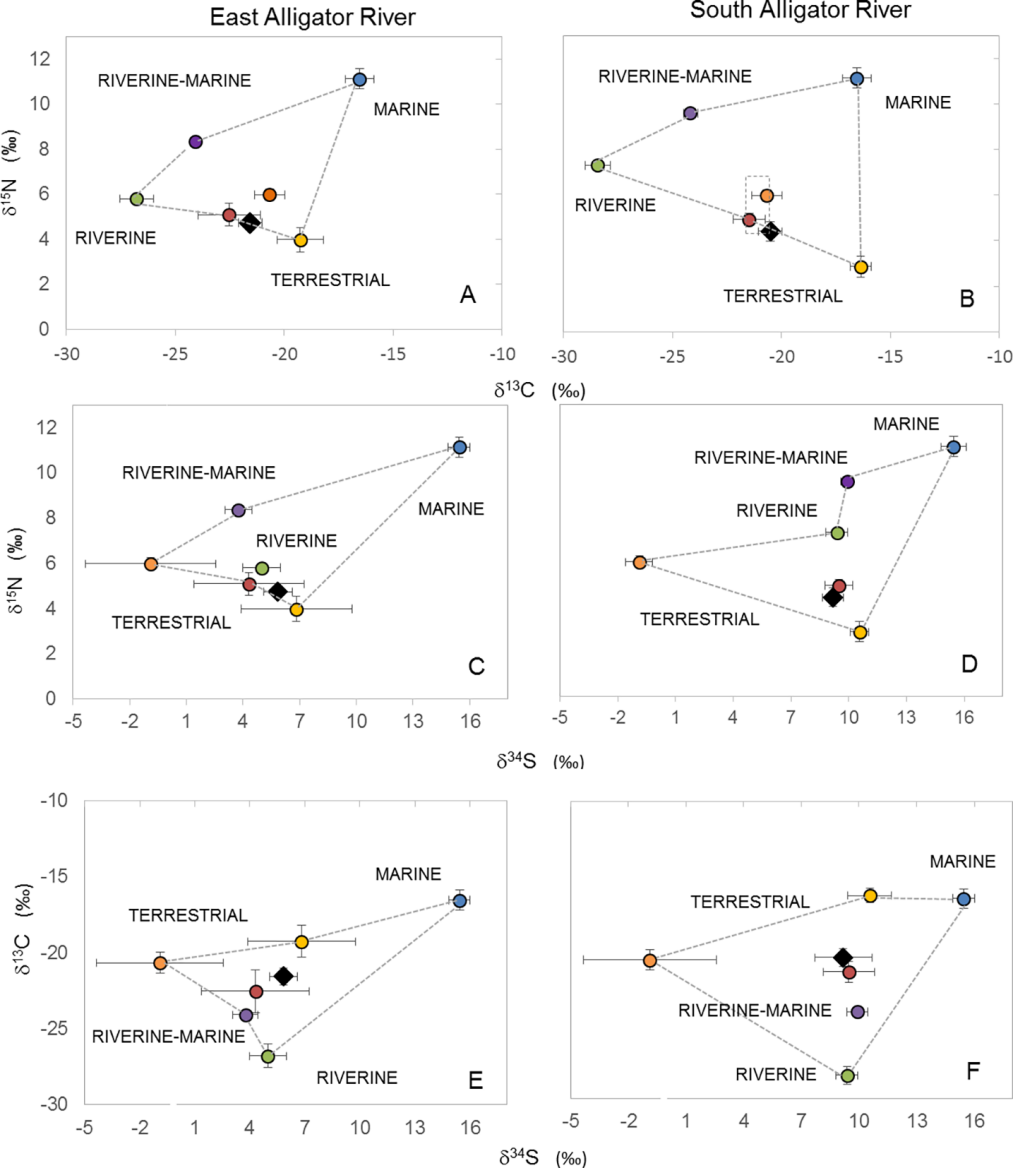
Isotopic composition (δ^34^S, δ^13^C and δ^15^N) of crocodiles (black diamonds) and potential prey. Samples were obtained from the East Alligator River (A,C, E) and South Alligator River (B,D,F), terrestrial prey includes water buffalo (orange circle), pigs (red circle) and wallabies (yellow circle); riverine is represented by mullet (green circle); riverine-marine prey is represented by barramundi (purple circle) and marine prey is represented by giant sea catfish (blue circle). Crocodile data were corrected to the level of prey by subtracting 1.4 ‰ from δ^13^C values [7]. The effect on the crocodile value of different fractionation factors is shown as a box of possible values around the crocodile mean in panel B.
