# Supplementary figures and images for: Estuarine crocodiles in a tropical coastal floodplain obtain nutrition from terrestrial prey
Source: PLoS One. 2018 Jun 6;13(6):e0197159. doi: 10.1371/journal.pone.0197159 (PMC5991389; doi:10.1371/journal.pone.0197159)

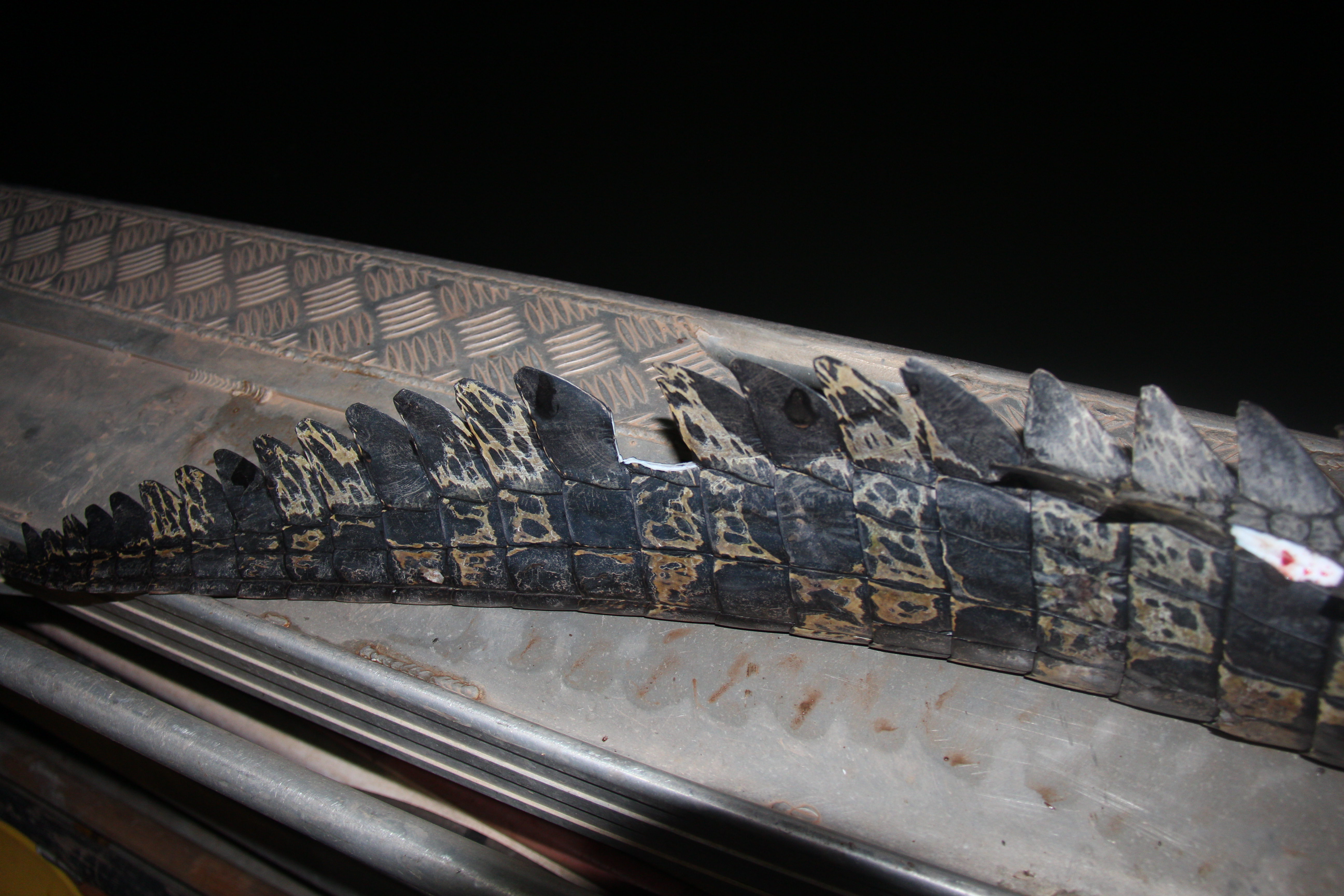

Supplement: S1 Fig — (TIFF) [file pone.0197159.s001.tiff]
