# Supplementary material for: Estuarine crocodiles in a tropical coastal floodplain obtain nutrition from terrestrial prey
Source: PLoS One. 2018 Jun 6;13(6):e0197159. doi: 10.1371/journal.pone.0197159 (PMC5991389; doi:10.1371/journal.pone.0197159)
Supplement: S1 Table — EAR = East Alligator River; SAR = South Alligator River, WR = Wildman River; M = Male; F = Female. (DOCX) [file pone.0197159.s002.docx]

**S1 Table.** Characteristics of *Crocodylus porosus* individuals from in Kakadu National Park, Australia. EAR= East Alligator River; SAR = South Alligator River, WR = Wildman River; M= Male; F= Female

| Number | Catchment | Site | Season | Date | Sex | Size (mm) |
| --- | --- | --- | --- | --- | --- | --- |
| 1 | EAR | Cahill's Crossing | Mid dry | 24/08/2012 | M | 2900 |
| 2 | EAR | Cahill's Crossing | Late wet | 30/05/2012 | M | 3800 |
| 3 | EAR | Cahill's Crossing | Late wet | 23/05/2012 | M | 2570 |
| 4 | EAR | Cahill's Crossing | Late dry | 25/09/2012 | M | 3070 |
| 5 | EAR | Cahill's Crossing | Late dry | 1/10/2012 | M | 3830 |
| 6 | EAR | Cahill's Crossing | Late wet | 16/05/2012 | F | 2940 |
| 7 | EAR | Cahill's Crossing | Late wet | 16/05/2012 | F | 2940 |
| 8 | EAR | Cahill's Crossing | Late wet | 16/05/2012 | M | 4200 |
| 9 | EAR | Cahill's Crossing | Mid dry | 24/08/2012 |  |  |
| 10 | EAR | Mudgimberri | Early wet | 17/01/2012 |  | 4000 |
| 11 | EAR | Mudgimberri | Early wet | 29/01/2012 |  | 4000 |
| 12 | EAR | Mudgimberri | Early wet | 27/02/2012 |  |  |
| 13 | EAR | Mudgimberri | Early wet | 27/01/2012 |  |  |
| 14 | EAR | Mudgimberri | Late wet | 9/03/2012 |  |  |
| 15 | EAR | Mudgimberri | Early wet | 12/02/2012 |  |  |
| 16 | EAR | Mudgimberri | Early wet | 26/01/2012 |  |  |
| 17 | EAR | Mudgimberri | Early wet | 2/01/2012 |  |  |
| 18 | EAR | Mudgimberri | Late Wet | 19/03/2012 |  |  |
| 19 | EAR | Cahill's Crossing | Late Dry | 29/10/2011 | M | 3030 |
| 20 | EAR | Cahill's Crossing | Late Wet | 25/04/2013 |  |  |
| 21 | EAR | Cahill's Crossing | Late Wet | 25/04/2013 | M | 2400 |
| 22 | EAR | Cahill's Crossing | Late Wet | 21/05/2013 | F | 1900 |
| 23 | EAR | Cahill's Crossing | Dry | 22/07/2013 | F | 2800 |
| 24 | EAR | Cahill's Crossing | Dry | 22/07/2013 | M | 2800 |
| 25 | EAR | Cahill's Crossing | Dry | 22/07/2013 | F | 2350 |
| 26 | EAR | Cahill's Crossing | Dry | 14/06/2013 |  |  |
| 27 | EAR | Cahill's Crossing | Dry | 12/06/2013 |  |  |
| 28 | EAR | Nankeen | Early wet | 10/02/2014 |  | 850 |
| 29 | EAR | Magela Crossing | Late Wet | 16/07/2014 |  | 1013 |
| 30 | EAR | Cahill's Upstream | Late Wet | 5/06/2014 |  | 2200 |
| 31 | EAR | Jabiluka Billabong | Late Wet | 19/06/2014 |  |  |
| 32 | SAR | River boat ramp | Late Dry | 18/09/2012 | M | 2920 |
| 33 | SAR | Nourlangie | Late Dry | 12/09/2012 | M | 4000 |
| 34 | SAR | Barramundi Gorge | Late Wet | 1/05/2012 |  |  |
| 35 | SAR | Barramundi Gorge | Late Wet | 17/05/2013 | M | 3900 |
| 36 | SAR | Jim Jim Falls | Late Wet | 14/05/2013 | M | 3600 |
| 37 | SAR | Jim Jim Falls | Dry | 14/06/2013 |  |  |
| 38 | SAR | Jim Jim Creek | Dry | 5/07/2005 |  |  |
| 39 | SAR | Jim Jim Creek | Dry | 5/07/2005 |  |  |
| 40 | SAR | Jim Jim Creek | Late Dry | 29/11/2006 |  |  |
| 41 | WR | Wildman River | Late Wet | 16/05/2012 | M | 4200 |
| 42 | WR | 4-Mile Hole | Late Dry | 19/09/2012 |  | 2690 |
| 43 |  | Unknown | Late Dry | 4/09/2012 |  |  |
| 44 |  | Unknown | Late Dry | 4/09/2012 |  |  |
| 45 |  | Unknown | Late Dry | 1/09/2012 |  |  |
