# Supplementary material for: Estuarine crocodiles in a tropical coastal floodplain obtain nutrition from terrestrial prey
Source: PLoS One. 2018 Jun 6;13(6):e0197159. doi: 10.1371/journal.pone.0197159 (PMC5991389; doi:10.1371/journal.pone.0197159)
Supplement: S2 Table — Values are mean ± standard error. (DOCX) [file pone.0197159.s003.docx]

**S2 Table.** Isotope values of potential prey for estuarine crocodiles (*Crocodylus porosus*) in Kakadu National Park, Australia. Values are mean ± standard error

| Habitat | Species | δ^13^C (‰) | δ^15^N (‰) | δ^34^S (‰) |
| --- | --- | --- | --- | --- |
| Terrestrial | Bandicoot  (*Isoodon macrourus**; n=1) | -20.0 | 5.1 | n.a. |
|  | Magpie geese  (*Anseranas semipalmata*; n =8) | -26.7 ± 0.5 | 7.4 ± 0.3 | 3.4 ± 3.2 |
|  | Goanna  (*Varanus sp*; n=2) | -18.7 ± 0.4 | 8.1 ± 1.5 | 10.4 ± 2.3 |
| Riverine | Pig nose turtle  (*Carettochelys insculpta*; n =2) | -26.1 ± 1.1 | 6.2 ± 0.2 | -0.3 ± 3.5 |
|  | Long neck turtle  (*Chelodina sp*; n = 2) | -25.5 ± 0.7 | 8.2 ± 0.1 | 5.0 ± 0.9 |
|  | Shrimp** | -20.5 ± 0.6 | 6.9 ± 0.3 | 16.2 |
|  | Crab** | -17.2 ± 0.4 | 7.2 ± 0.5 | n.a. |
|  | Snail** | -17.2 ± 0.3 | 6.0 ± 0.6 | n.a. |
| Riverine-marine | Bullshark  (*Carcharhinus leucas*; n=9) | -19.1 ± 0.9 | 10.9 ± 0.4 | 11.9 ± 0.6 |
|  | Northern River Shark  (*Glyphis garricki*; n =10) | -19.2 ± 0.3 | 8.7 ± 0.2 | n.a. |

*most likely species

**multiple species
